# The Relationship Between Endorsement of the Sexual Double Standard and Sexual Cognitions and Emotions

**DOI:** 10.1007/s11199-016-0616-z

**Published:** 2016-04-08

**Authors:** Peggy M. J. Emmerink, Regina J. J. M. van den Eijnden, Ine Vanwesenbeeck, Tom F. M. ter Bogt

**Affiliations:** 1 Department of Interdisciplinary Social Sciences, Utrecht University, Martinus J. Langeveld Building, Room H2.20, Heidelberglaan 1, 3584 CS Utrecht, The Netherlands; 2 Rutgers, Expert Centre for Sexual and Reproductive Health and Rights, Utrecht, The Netherlands

**Keywords:** Sexuality, Emerging adulthood, Gender roles, Sexual attitudes, Social norms

## Abstract

Sexual gender norms promoting sexual prowess for men, but sexual modesty for women have been shown to negatively affect sexual and mental health in both men and women. Knowledge about the relationship between gender norms and sexual cognitions and emotions might further the understanding of continued gender-norm conformity. In the present study, we investigated whether and how gendered sexual attitudes are related to (positive and negative) sex-related emotions, via sexual cognitions (autonomy, body esteem, and approach/avoidance motives for sex). A survey was submitted to 293 heterosexually identified, sexually active, Dutch (central and southern regions) 18–25 year-olds. Results from a moderated mediation model (using structural equation modeling) revealed that women, but not men, who more strongly endorsed traditional gender-related sexual attitudes experienced significantly less positive and more negative emotions, as a result of decreased sexual autonomy and sexual body esteem as well as increased avoidance motives for sex. We conclude that, as a result of negative cognitions associated with the endorsement of traditional gender norms, women in particular are at risk of experiencing negative emotional outcomes in the sexual context. We argue that sexual cognitions and emotions deserve explicit attention in sex-positive and gender-transformative sexuality education, which has been proven to be a vital resource for achieving increased gender-equity in sexual and romantic relationships.

**Electronic supplementary material:**

The online version of this article (doi:10.1007/s11199-016-0616-z) contains supplementary material, which is available to authorized users.

Traditional gender norms promoting sexual modesty for girls and women, but sexual prowess for boys and men have been recognized in numerous cultures across the globe. This gendered pattern, often referred to as the *sexual double standard*, generally implies that men are expected to present themselves as sexually active and ready to take sexual initiatives, whereas women are expected to present themselves as sexually reactive and passive (Bordini and Sperb 2013; Crawford and Popp 2003; Sanchez et al. 2012a; Vanwesenbeeck 2009). Gender-related sexual attitudes in line with the sexual double standard have been related to reduced sexual and mental health in numerous studies. A recent literature review, which included accounts of adolescents and (young) adults in the United States, concluded that although both men’s and women’s health is affected, the submissive role played by women may be particularly harmful (Sanchez et al. 2012a).

Examples of harmful effects found among U.S. samples include links between traditional gender-related sexual attitudes and increased rape myth acceptance among men (Truman et al. 1996), poor sexual functioning among emerging adults (Kiefer and Sanchez 2007), increased sexual problems among young adult and adult women (Sanchez and Kiefer 2007), and reduced sexual and relationship satisfaction among young adults (Sanchez et al. 2005). Although studies have primarily taken place in the United States (Bordini and Sperb 2013; Crawford and Popp 2003), effects on sexual and mental health are found in diverse samples throughout the world. Sexual double standards have, for example, been associated with increased dating and sexual violence among Chinese adolescents (Shen et al. 2012), with early sexual initiation among Estonian adolescents (Part et al. 2011) and young adults in Southern Brazil (Goncalves et al. 2008), and with a higher risk for STI/HIV infection among Spanish adolescents (Bermúdez et al. 2010).

The majority of studies on the sexual double standard have focused heavily on the analysis of its detrimental effects on sexual health (Bordini and Sperb 2013). Although it is important to investigate these health effects, we believe it is equally important to understand the cognitive (i.e. sexual autonomy, sexual motives, and body esteem) and emotional (i.e. positive and negative sex-related affect) repercussions of conformity to gender norms. As Sanchez et al. (2012a) suggested, this psychosexual domain has received insufficient attention in previous studies, but it could nonetheless provide an insight into the continued influence of gender-norm conformity in sexuality. The scarcity of research on the relationships between gender-related sexual attitudes and sexual self-related cognitions and emotions, while negative associations between traditional gender-related sexual attitudes and sexual health are ubiquitous, constitutes a gap in the literature. In the present study, we accordingly aim to investigate this relationship.

Positive and negative affect can be presumed to play an important role in people’s lived experiences concerning sexuality in general. Emotions (as well as cognitions) in sexual situations can be both the result and the instigator of sexual behavior. Because we know that traditional gender-related sexual attitudes negatively affect women in particular (Sanchez et al. 2012a), this could imply that women’s emotions surrounding sexuality might also be more negative. This proposal is related to findings of other studies from which we know that women tend to experience less positive and more negative sex-related affect compared to men, for whom the experience appears to be more uniformly positive (De Graaf et al. 2012; Geer and Robertson 2005; Impett and Tolman 2006). From studies on affective reactions to first-time sexual intercourse, we also know that women are more likely to report negative affect, such as shame and guilt, than are men (De Graaf et al. 2012; Vasilenko et al. 2015).

Although many different factors may be responsible for these gender differences, women presumably experience more negative and less positive sex-related affect compared to men, (partly) as a function of gender-norm conformity. Furthermore, this can be presumed to happen through sexual self-related cognitions. We know from the literature on attentional and information processes in producing sexual arousal that cognitions and emotions work together, following a dual pathway model discerning a more automatic (reflexive) and a more deliberate pathway (reflective). Moreover, this research highlights the relevance of deliberate rather than automatic cognitive processes (Brauer et al. 2009). A better understanding of the cognitive and affective processes associated with gender-related sexual attitudes not only could clarify its influence on the formation and expression of men’s and women’s sexual selves, but also might eventually help explain the adverse health effects that are generally related to these attitudes. Moreover, investigation of the aforementioned assumption might advance our understandings as to why women, as compared to men, tend to experience more sexual dysfunction (Laumann et al. 1999) as well as lower levels of satisfaction in sexual relationships (Petersen and Hyde 2010).

Therefore, both positive and negative affect, as well as cognitive factors, are presumed to play an important role in informing sexual behavior (and vice versa). In our study, we examined four cognitive factors that, based on the literature, could be proposed to mediate the relationship between gender-related sexual attitudes and positive and negative affect in the sexual context. Because various studies find increased sexual autonomy and assertiveness among men and more compliance and inauthenticity in relationships among women (Impett et al. 2006), sexual autonomy is an interesting cognitive factor mediating the link between attitudes and affect. *Sexual autonomy* is defined as the general sense of control that individuals experience and the extent to which they feel unburdened by external pressures in sexual situations (Sanchez et al. 2006). One previous study found decreased sexual autonomy to be positively related to pressure towards gender conformity for women but negatively related to this pressure for men (Sanchez and Kiefer 2007). Likewise, another study found increased sexual submissiveness among women to be related to decreased sexual autonomy, which in turn predicted lower sexual arousal (Sanchez et al. 2006). Although positive and negative sex-related affect is by no means synonymous with sexual arousal or pleasure, we would expect them to be influenced in a similar way. We, therefore, hypothesize that stronger endorsement of traditional gender-related sexual attitudes will be related to lower levels of autonomy in women and thus to lower levels of positive and higher levels of negative sex-related affect. Conversely, we hypothesize that stronger endorsement of traditional gender-related sexual attitudes will be related to higher levels of autonomy in men and thus to more positive and less negative sex-related affect.

A second cognitive factor that might mediate the relationship between gender-related sexual attitudes and sex-related affect is sexual body esteem. Sexual body esteem has previously been associated with resistance to the sexual double standard in a sample of Australian adolescent women (Horne and Zimmer-Gembeck 2005). In the same study, sexual body esteem was also positively related to happiness (Horne and Zimmer-Gembeck 2005). Moreover, sexual body esteem is conceptually linked to sexual subjectivity. Subjectivity requires an understanding and experience of sexual pleasure, which is in turn less likely if individuals objectify their sexual selves and allow others to judge them (Horne and Zimmer-Gembeck 2006). Objectification theory posits that women’s internalized observer’s perspective leads to self-objectification and habitual body monitoring, which, in turn, can increase women’s opportunities for shame and anxiety. And because women are acutely aware of their outer body appearance, they may be left with fewer perceptual resources available for attending to inner body experience, greatly hindering their sexual satisfaction (Fredrickson and Roberts 1997). Because traditional gendered norms prescribe that women should be physically attractive and sexy, but not sexual (as related to the Madonna-Whore dichotomy or the Virgin-Slut Continuum), we expect that increased traditional gender-related sexual attitudes will be related to decreased sexual body esteem. In turn, we expect both of these to be associated with less positive and more negative sex-related affect. Because much less is known on this topic for men, and objectification seems to be a problem that is more prevalent among women, we hypothesize that the link between gender-related sexual attitudes and sexual body esteem will be much weaker for men than for women, but will be similarly related to other variables in the model.

Thirdly and fourthly, approach and avoidance motives, concepts that have been used in the field of psychology for decades, are relevant correlates to take into consideration. Motivation seems to be regulated by two systems that either dictate behavioral activation or behavioral inhibition, and individuals can differ in their relative sensitivity (Gray 1987), making some people especially responsive to reward cues, whereas others are particularly responsive to threat and punishment cues (Carver and White 1994). Approach and avoidance social goals are cognitive representations that respectively direct individuals towards potentially positive relational outcomes or away from potentially negative ones (Elliot et al. 2006). In the sexual context, approach motives focus on securing positive outcomes, such as personal physical pleasure and enhanced partner intimacy, whereas avoidance motives focus on the prevention of negative outcomes, such as a partner getting angry or losing interest in the relationship (Impett et al. 2005).

It appears that approach and avoidance tendencies concerning sexuality are at least partially learnt through gendered socialization in childhood and adolescence during which girls are faced with the complicated ambiguity of having to reconcile pursuing pleasure (e.g., a satisfying sex life) and avoiding risk (e.g., of stigma or crossing of sexual boundaries) (Tolman 2006). For boys, gendered socialization concerning sexuality seems to be much less ambiguous; they are pulled in a generally more pro-sex direction (Shibley-Hyde and Durik 2000). On the basis of these differences in gendered socialization, we would expect both approach and avoidance motives to play a role for women, whereas for men, avoidance motives may play a less prominent role. Previous research tells us that general approach tendencies in romantic relationships strengthen the well-being and success of a relationship, whereas avoidance tendencies are negatively associated with relationship quality (Frank and Brandstätter 2002; Impett et al. 2005). Accordingly, we expect these tendencies to be related in a similar manner to positive and negative affect, respectively.

We, therefore, argue that approach and avoidance sexual motives constitute an important cognitive correlate of gender-normative sexual attitudes, which could in turn influence positive and negative sex-related affect. In line with the sexual double standard, we hypothesize that stronger endorsement of traditional gender-related sexual attitudes will be related to lower approach and higher avoidance motives in women, and thus to lower positive and higher negative sex-related affect. For men, however, we hypothesize that more traditional gender-related sexual attitudes will be related to higher approach and lower avoidance motives for sex, and thus to more positive and less negative sex-related affect.

In summary, we investigated the relationship between gender-related sexual attitudes and positive and negative sex-related affect, mediated by cognitive factors (i.e. sexual autonomy, sexual body esteem, approach and avoidance motives for sex). This relationship is examined with a sample of Dutch emerging adults. We chose to examine gender-related sexual attitudes in emerging adulthood because this is when explorations of love become more intimate and serious (Arnett 2000). Additionally, emerging adults from the Netherlands seem to provide an interesting sample. The country has an image of being more open-minded about (adolescent) sexuality in comparison to other countries, and the Dutch government supports comprehensive sexuality education (Dodge et al. 2005). Comprehensive sexuality education programs in the Netherlands are aimed at teaching young people about sexual responsibility, and they include messages on sexual assertiveness and on “only doing what you want to do” regardless of whether one is male or female (Ferguson et al. 2008). Based on this information, we could expect that traditional gendered attitudes regarding sexuality might be less of a problem in the Netherlands.

This loosing of the sexual double standard appears to be reflected in research to some extent: an initial Dutch study on the topic found that, on average, Dutch young people endorse the notion of gender equality more than that of the sexual double standard (Emmerink et al. 2016). However, the same study also showed that there are still (groups of) individuals that support different sexual standards for men and women. Being open-minded about sex and incorporating comprehensive sexual education, therefore, does not mean that normative gender roles in line with the sexual double standard do not exist and may even mean that the problem exists in a more covert form.

Given the nature of the concepts we measured in the present study, we wished to include sexually active participants only. The fact that relationships among emerging adults are more likely to include sexual intercourse than relationships among adolescents (Arnett 2000) was therefore a contributory factor in our sample choice. Although we do not seek to imply that the phenomenon studied does not affect non-heterosexual participants, our study further focused on heterosexual individuals. Lastly, although a focus on gender similarities, rather than on differences, is to be preferred in research in general (Hyde 2005), the sexual context is one in which both perspectives arguably have advantages. On the one hand, focusing on differences must not be a decision that is taken lightly, because it might contribute to double standards and lead to the overstating of gendered sexual differences (Hyde 2005; Vanwesenbeeck 2009). On the other hand, focusing on similarities may disguise real differences concerning sexuality in the population, thus foregoing the possibility of advancing knowledge on the topic (Vanwesenbeeck 2009). Because the sexual double standard is by definition a gendered concept and because gender differences found in previous research justify this approach, we take a gender differences perspective in our study.

The overarching research question addressed in our study is: To what extent are gender-related sexual attitudes associated with positive and negative sex-related affect through self-related sexual cognitions (sexual autonomy, sexual body esteem, and approach / avoidance motives for sex) among heterosexually identified, sexually active, emerging adult men and women in the Netherlands? We addressed this question using a structural equation modeling approach (Kline 1998) to test a multi-group moderated (gender) mediation model. Although we chose this particular set-up to illustrate the relationship among gender-related sexual attitudes, cognitions, and emotions, these variables are presumed to be in continuous interaction with each other. Cognitions are able to influence emotions, but emotions are also able to influence cognitions. Similarly, activated cognitions and emotions in sexual situations may be both the result and the instigator of sexual behavior, and they may enforce or weaken sexual attitudes. We see this process as an example of “doing gender” (West and Zimmerman 1987, p. 126), which posits gendered behavior as a “situated doing, carried out in the virtual or real presence of others who are presumed to be oriented to its production” and as a day-to-day enactment of prescribed social roles and the sexual double standard.

With this research design, we hope to be able to shed light on cognitive and emotional processes with regard to gender-related sexual attitudes, without necessarily implying one-directional causal relationships. Current knowledge suggests two general hypotheses. (a) Women’s stronger endorsement of traditional gender-related sexual attitudes will be related to lower levels of positive and higher levels of negative sex-related affect through lowered sexual autonomy, sexual body esteem, and approach motives for sex, and heightened avoidance motives for sex (Hypothesis 1). (b) Men’s stronger endorsement of traditional gender-related sexual attitudes will be related to higher levels of positive and lower levels of negative sex-related affect through heightened sexual autonomy and approach motives for sex, but lower avoidance motives for sex (Hypothesis 2).

## Method

### Participants and Procedure

Participants were asked to complete an online questionnaire on sexuality and dating. Participants were recruited on campus by six graduate research assistants who handed out flyers and put up posters with a link to the questionnaire, by sending out e-mails and sharing the questionnaire link on social media within personal networks, and by asking others to forward e-mails and share links. Participants received no monetary compensation for their participation, but were eligible to win a gift voucher. To increase the response and sharing of the link, it was truthfully stated in the invitation that the more participants filled in the questionnaire, the more gift vouchers would be allocated.

The original sample consisted of a convenience sample of 351 emerging adults living in the Netherlands (central and southern regions of the country). Participants were excluded if they indicated that they were attracted exclusively or mainly to members of their own sex, were attracted to both sexes equally or were undecided about their sexual orientation. This screening resulted in the exclusion of 14 participants.

Of the remaining 337 emerging adults in the sample, 293 (86.9 %) indicated that they had been sexually active at some point. Because we also asked questions relating to personally experienced sexual behavior (i.e. sexual autonomy, approach and avoidance motives, and affect experienced after sex), we only included sexually active participants. The largest proportion of the final sample of 293 sexually active participants consisted of native Dutch men (*N* = 117, 92.9 %) and native Dutch women (*N* = 150, 89.8 %). Of the male participants 112 (88.9 %) and of the female participants 146 (87.8 %) indicated that they were non-religious. As can be expected based on the included age range of participants in the study, a large proportion of them were students at various stages of their education (*n* = 208, 71.0 %). The remainder of participants were currently in paid employment (*n* = 78, 26.6 %) or did not indicate their current occupation (*n* = 7, 2.4 %). The largest proportion of participants were either in or had completed higher education (pre-university education or university: 59 men (46.8 %), 107 women (64.5 %), followed by intermediate education (intermediate general education or vocational training: 50 men (39.7 %), 50 women (30.1 %), and lower education (primary school and junior vocational training: 17 men (3.5 %), 9 women (5.4 %). The final sample for analysis consisted of 293 heterosexual, sexually active, emerging adults (167 women, 57.0 %) aged 18 to 25 (*M*_men_ = 22.01, *SD* = 2.14; *M*_women_ = 21.93, *SD* = 1.98).

### Materials

The questionnaire was made available at an online location. Participants were asked to go to this online location on their own personal computer. An introduction page instructed participants that the study would be on sexuality and dating and that should they have any questions, they could address these to the first author’s e-mail address. Participants were ensured that their responses would be anonymous and that they could cease their participation at any time. They were asked to fill in the questionnaire as honestly as possible and instructed that there were no right or wrong answers. Participants filled in the measures in the order in which they appear below. A definition of sex was offered to participants before answering the questions on sexual cognitions and emotions and gender-related sexual attitudes: “By ‘sex’ we mean everything from feeling each other naked or caressing each other, to intercourse (penetration of the vagina or anus by the penis).” It took participants about 20 min to complete the questionnaire. After filling in the questionnaire participants were debriefed through e-mail and again offered contact information of the researchers should they have any questions. The overall number of missing values was very low (1.7 %) and assessed to be missing completely at random. Missing values were imputed using the expectation maximization procedure in IBM SPSS 22©.

#### Demographics

Participants indicated their age in years, their gender, and their ethnicity. This last variable is important because the Netherlands is a multicultural society and any sample description from a Dutch study should therefore include ethnic background. In line with Dutch guidelines, ethnicity was based on both parents’ countries of birth (Statistics Netherlands 2012). If one or both parents of an individual were not born in the Netherlands, that individual is generally referred to as non-ethnically Dutch (Statistics Netherlands 2012). These participants do hold Dutch nationality, but have a (partly) non-Dutch cultural background. Additionally, participants answered a question on religion. Lastly, participants indicated their current education (or educational aspirations)*.* If participants indicated that their most important occupation was studying, the education they were following was taken as their education level. If participants indicated that they were no longer studying, the highest-level academic qualification that they had attained was taken as their education level.

#### Gender-Related Sexual Attitudes

To assess gender-related sexual attitudes we used the Scale for the Assessment of Sexual Standards Among Youth (SASSY) (Emmerink et al. 2016). This scale measures “the degree to which an individual’s attitude reflects a divergent set of expectations for men and women, in that men are expected to be relatively more sexually active, assertive and knowledgeable and women are expected to be relatively more sexually reserved, passive and inexperienced” (p. 4). The instrument consists of 19 statements about men and women, about which participants indicated their degree of agreement on a 6-point scale ranging from 1 (*completely disagree*) to 6 (*completely agree*). The scale was submitted to respondents in the Dutch language. The exact item wording in Dutch, as well as the English translation, can be found in an online supplement. Two sample items are: “Girls should act in a more reserved way concerning sex than boys” [Meisjes moeten zich op het gebied van seks terughoudender gedragen dan jongens]” and “It is more important for a girl to look attractive than it is for a boy” [“Voor een meisje is het belangrijker om er aantrekkelijk uit te zien dan voor een jongen”]. An introduction to the instrument explained that when items mentioned boys and girls, they were also intended to include men and women. The SASSY yielded good reliability in a previous study, with a Cronbach’s alpha of .90 (Emmerink et al. 2016). In the present study we obtained a Cronbach’s alpha of .88. For use in the analyses, we calculated a mean score on the 19 items such that higher scores indicated greater endorsement of gender-related sexual attitudes.

#### Sexual Autonomy

Sexual autonomy was assessed using a six-item scale adapted from other instruments (Sanchez et al. 2012b; Vanwesenbeeck et al. 2003). Translations of the six items, each completing the stem “If I am in a sexual interaction with someone I really like…,” are: “…I like to be dominant,” “…I tend to take the initiative,” “…I tend to take an active role,” “…I know exactly what I want,” “…I can determine what happens and voice my opinion,” and “…I feel free to be who I am.” Participants indicated on a scale ranging between 1 (*not at all*) to 6 (*very well*) the extent to which the items described them personally. In the present study, we obtained a Cronbach’s alpha of .81 for this scale. For use in the analyses, we calculated a mean score on the six items such that higher scores indicated greater sexual autonomy.

#### Sexual Body Esteem

Sexual body esteem was assessed by indicating the extent of agreement with five items, such as: “I am confident that a romantic partner would find me sexually attractive.” Answers ranged from 1 (*completely disagree*) to 6 (*completely agree*). The items composed a subscale (Element 1) of the Female Sexual Subjectivity Inventory (Horne and Zimmer-Gembeck 2006). In our study, we obtained a Cronbach’s alpha of .86 for this scale. For use in the analyses, we calculated a mean score on the five items such that higher scores represented greater sexual body esteem.

#### Approach Motives for Sex

Approach motives for sex were defined as motives that focus on (personal or partner) desire, love, and interpersonal intimacy. The items were taken from a previous study (Impett et al. 2005). A sample item is: “If I have sex, I do so because I want to show love for my partner.” Participants indicated their agreement with five items on a scale ranging from 1 (*completely disagree*) to 6 (*completely agree*). In our study, we obtained a Cronbach’s alpha of .76 for this scale. For use in the analyses, we calculated a mean score on the five items such that higher scores captured higher approach motives for having sex.

#### Avoidance Motives for Sex

Avoidance motives for sex were defined as motives that focus on avoiding relationship conflict and preventing the partner from losing interest. The items were taken from a previous study (Impett et al. 2005). A sample item is: “If I have sex, I do so because I want to avoid conflict in my relationship.” Participants indicated their agreement to four items on a scale ranging from 1 (*completely disagree*) to 6 (*completely agree*). In our study, we obtained a Cronbach’s alpha of .73 for this scale. For use in the analyses, we calculated a mean score on the four items such that higher scores indicated higher motives for avoiding having sex.

#### Positive Sex-Related Affect

Positive sex-related feelings were assessed by asking participants: “How do you feel when you have just had sex?” Participants indicated on a scale ranging between 1 (*not at all*) to 6 (*very well*) the extent to which the emotions presented described their feelings after sex. The scale comprised three items: “feeling proud,” “feeling happy,” and “feeling loved.” These items were adapted for use in the current study from previously used instruments (Schwartz 1993; Tracy et al. 2003). In our study, we obtained a Cronbach’s alpha of .76 for this scale. For use in the analyses, we calculated a mean score on the three items wherein higher scores indicated stronger positive sex-related affect.

#### Negative Sex-Related Affect

Negative sex-related feelings were assessed by asking participants: “How do you feel when you have just had sex?” Participants indicated on a scale ranging between 1 (*not at all*) to 6 (*very well*) the extent to which the emotions presented described their feelings after sex. The scale comprised three items: “feeling ashamed,” “feeling dirty,” and “feeling guilty.” These items were adapted for use in the current study from previously used instruments (Schwartz 1993; Tracy et al. 2003). In our study, we obtained a Cronbach’s alpha of .83 for this scale. For use in the analyses, we calculated a mean score on the three items so that higher scores represented stronger negative sex-related affect.

## Results

### Descriptive Statistics and Correlations

Mean scores for men and women on gender-related sexual attitudes and sexual cognitions and emotions were compared using an overall MANOVA (see Table 1). No significant differences in mean scores were found between men and women on gender-related sexual attitudes. Among the cognitive and affective correlates, only sexual autonomy was significantly different between men and women, with men scoring significantly higher than women.

**Table 1:** Mean differences between men and women

Variable	Men (*n* = 126)		Women (*n* = 167)		*F* (1291)
	*M*	*SD*	*M*	*SD*	
Gender-related sexual attitudes	2.38	.69	2.23	.62	3.86
Sexual autonomy	4.15	.76	3.87	.72	10.62**
Sexual body esteem	4.16	.98	4.12	.94	.10
Approach motives for sex	4.82	.64	4.74	.66	1.22
Avoidance motives for sex	2.32	.92	2.16	.95	2.43
Positive feelings after sex	5.00	.62	4.95	.66	.57
Negative feelings after sex	1.69	.73	1.75	.92	.27

All measures used scales from 1 to 6. Higher scores indicate more endorsement of traditional gender-related sexual attitudes, higher scores on sexual autonomy, sexual body esteem, approach motives for sex, avoidance motives for sex, positive feelings after sex and negative feelings after sex

** *p* < .01

Looking at bivariate Pearson correlations between the variables in the proposed model for men (see Table 2), stronger traditional gender-related sexual attitudes were significantly related only to avoidance motives for sex. Additionally, increased positive and decreased negative sex-related affect was significantly related to increased sexual autonomy, sexual body esteem, and approach motives for sex among men.

**Table 2:** Correlations among study variables

Variables	1.	2.	3.	4.	5.	6.	7.
1. Gender-related sexual attitudes	–	−.289**	−.235**	−.016	.442**	−.124	.310**
2. Sexual autonomy	.136	–	.417**	.211**	−.243**	.323**	−.368**
3. Sexual body esteem	−.090	.471**	–	.174*	−.201**	.306**	−.305**
4. Approach motives for sex	−.069	.239**	.093	–	.112	.537**	−.137
5. Avoidance motives for sex	.171*	−.218**	−.283**	.025	–	−.101	.289**
6. Positive feelings after sex	.028	.395**	.228**	.432**	−.062	–	−.350**
7. Negative feelings after sex	.061	−.285**	−.373**	−.176*	.442**	−.218**	–

Correlations for women are reported above the diagonal; for men, below. Higher scores indicate increased endorsement of traditional gender normative sexual attitudes, higher scores on sexual autonomy, sexual body esteem, approach motives for sex, avoidance motives for sex, positive feelings after sex and negative feelings after sex

*p < .05. **p < .01

For women, stronger traditional gender-related sexual attitudes were significantly related to decreased sexual autonomy, decreased sexual body esteem, increased avoidance motives for sex, and increased negative sex-related affect (see Table 2). Additionally, increased positive and decreased negative sex-related affect was significantly related to increased sexual autonomy and increased sexual body esteem among women. Furthermore, positive sex-related affect was significantly positively related to approach motives for sex (but not to avoidance motives), whereas negative sex-related affect was significantly positively related to avoidance motives for sex (but not to approach motives).

### The Moderated Mediation Model

**Fig. 1:**
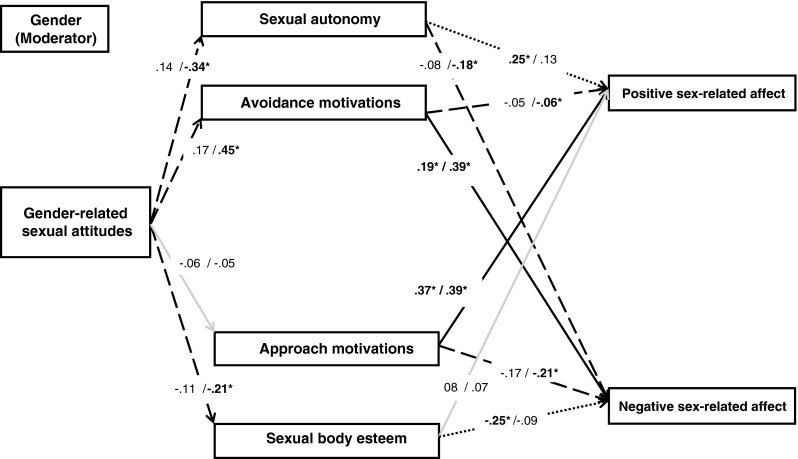
A graphical representation of the multi-group moderated mediation model for men (regression weights reported before the slash) / women (after the slash). *Solid black lines* indicate significant paths for both men and women at *p* < .05. *Dotted black lines* indicate a significant path for men only at *p* < .05. *Dashed black lines* indicate a significant path for women only at *p* < .05. *Grey lines* indicate non-significant paths for both men and women. *p < .05

The multi-group (moderated) mediation model presented in Fig. 1 was tested using structural equation modeling in AMOS (Version 7.0; Arbuckle 2006). The relationship between gender-related sexual attitudes with positive and negative sex-related affect was modeled through the cognitive mediators of sexual autonomy, sexual body esteem, and approach and avoidance motives for sex. Gender was included as a moderator. We used a bootstrap procedure to estimate models in order to be able to confirm mediation effects. Bootstrapping simultaneously alleviated a minor analysis problem because the variable of negative sex-related affect was profoundly left-skewed (Efron and Tibshirani 1994). We obtained 1000 bootstrap samples and analyzed the 95 % bias-corrected confidence intervals (95 % CI; unstandardized values for these confidence intervals are presented) for all hypothesized effects. A relationship in the model can be considered statistically significant when the corresponding confidence interval does not include zero.

The initial model test provided modification indices suggesting some additional pathways (correlated error terms for sexual autonomy and approach motives, for sexual autonomy and sexual body esteem, for sexual body esteem and avoidance motives, and for positive and negative affect), which were included in the model and further improved the fit. For this final model, model fit was evaluated and considered adequate with *χ*^2^(10) = 15.83, *p* > .05, a Comparative Fit Index (CFI) of .98 (> .90), and a Root Mean Square Error of Approximation (RMSEA) of .05 (< .08; Kline 1998).

Additionally, an identical model to that presented in Fig. 1 was tested, but for two additional direct arrows from gender-related sexual attitudes to positive and negative sex-related affect. This model did not show adequate fit (RMSEA > .10), even when adding additional pathways as suggested under the modification indices (correlated error terms). This suggests that gender-related sexual attitudes are related to sex-related affect *through* self-related cognitions. Lastly, we also estimated identical models including the control variables of age and ethnicity. Neither variable added to the model fit, nor did they significantly change other relationships that were found between variables in the model.

#### Direct Relationships Between Variables for Men

We evaluated the direct relationships for men using 95 % bias-corrected confidence intervals through bootstrapping. For men, stronger traditional gender-related sexual attitudes were not significantly related to sexual autonomy (*B* = .151, *β* = .137, *p* = .121, 95 % CI [−.070, .462]), sexual body esteem (*B* = −.147, *β* = −.105, *p* = .238, 95 % CI [−.443, .121]), approach motives for sex (*B* = −.051, *β* = −.055, *p* = .535, 95 % CI [−.218, .118]) or avoidance motives for sex (*B* = .220, *β* = .165, *p* = .062, 95 % CI [−.004, .462]). Increased sexual autonomy was also not significantly related to either negative sex-related affect (*B* = −.075, *β* = −.078, *p* = .378, 95 % CI [−.283, .159]) or positive sex-related affect (*B* = .203, *β* = .250, *p* = .003, 95 % CI [.012, .413]). Increased sexual body esteem was significantly related to decreased negative sex-related affect (*B* = −.188, *β* = −.250, *p* = .005, 95 % CI [−.316, −.046]), but was not significantly related to positive sex-related affect (*B* = .048, *β* = .075, *p* = .378, 95 % CI [−.061, .157]). Increased approach motivation was significantly related to both increased positive sex-related affect (*B* = .345, *β* = .369, *p* < .001, 95 % CI [.205, .501]) and decreased negative sex-related affect (*B* = −.197, *β* = −.173, *p* = .035, 95 % CI [−.389, −.026]). Lastly, increased avoidance motivation was significantly related to decreased positive sex-related affect (*B* = −.031, *β* = −.046, *p* = .560, 95 % CI [−.133, .079]) and increased negative sex-related affect (*B* = .152, *β* = .193, *p* = .021, 95 % CI [.005, .313]).

#### Direct Relationships Between Variables for Women

We also evaluated the direct relationships for women using 95 % bias-corrected confidence intervals through bootstrapping. For women stronger traditional gender-related sexual attitudes were significantly related to decreased sexual autonomy (*B* = −.379, *β* = −.342, *p* < .001, 95 % CI [−.607, −.202]), decreased sexual body esteem (*B* = −.312, *β* = −.208, *p* = .006, 95 % CI [−.533, −.070]), and increased avoidance motives for sex (*B* = .689, *β* = .453, *p* < .001, 95 % CI [.401, .929]), but were not significantly related to approach motives for sex (*B* = −.058, *β* = −.054, *p* = .483, 95 % CI [−.262, .149]). In turn, increased sexual autonomy was significantly related to decreased negative sex-related affect (*B* = −.228, *β* = −.180, *p* = .009, 95 % CI [−.403, −.016]), but not significantly related to positive sex-related affect (*B* = .116, *β* = .128, *p* = .081, 95 % CI [−.029, .271]). Increased sexual body esteem was not significantly related to negative sex-related affect (*B* = −.084, *β* = −.086, *p* = .211, 95 % CI [−.253, .092]) or positive sex-related affect (*B* = .051, *β* = .073, *p* = .315, 95 % CI [−.056, .164]). Increased approach motivation was significantly related to both increased positive sex-related affect (*B* = .389, *β* = .393, *p* < .001, 95 % CI [.169, .590]) and decreased negative sex-related affect (*B* = −.295, *β* = −.213, *p* = .001, 95 % CI [−.490, −.142]). Lastly, increased avoidance motivation was significantly related to decreased positive sex-related affect (*B* = −.044, *β* = −.063, *p* = .370, 95 % CI [−.161, .056]) and increased negative sex-related affect (*B* = .379, *β* = .391, *p* < .001, 95 % CI [.214, .595]).

#### Indirect Relationships Between Variables for Men and Women

We also evaluated the indirect effects using 95 % bias-corrected confidence intervals through bootstrapping. For women, the indirect effects of gender-related sexual attitudes on both positive sex-related affect (*B* = −.115, *β* = −.109, *p* = .045, 95 % CI [−.242, −.003]) and negative sex-related affect (*B* = .395, *β* = .268, *p* = .001, 95 % CI [.223, .596]) were significant. This confirms Hypothesis 1, which predicted that stronger endorsement of traditional gender-related sexual attitudes would be related to decreased positive and increased negative sex-related affect indirectly through cognitive factors for women. When taking the results for the direct paths into account, we find that Hypothesis 1 is confirmed only for sexual autonomy and avoidance motives for sex.

For men, neither the indirect effect of gender-related sexual attitudes on positive sex-related affect (*B* = −.001, *β* = −.002, *p* = .997, 95 % CI [−.099, .113]) nor on negative sex-related affect (*B* = .060, *β* = .057, *p* = .283, 95 % CI [−.057, .177]) was significant. Therefore, Hypothesis 2, which posited that stronger endorsement of traditional gender-related sexual attitudes would be related to decreased positive and increased negative sex-related affect indirectly through cognitive factors for men, has to be rejected.

#### Significance of Gender Differences

Because the moderated mediation model was tested as a multi-group (men and women) model, we can conclude that the results represent true differences in the relationship between gender and gender-related sexual attitudes.

## Discussion

The aim of our study was to investigate associations between gender-related sexual attitudes and self-related sexual cognitions and emotions. Traditional gender-related sexual attitudes were found to be associated with decreased sexual autonomy and increased avoidance motives for sex, which were in turn related to decreased positive, and increased negative, affect among women. However, among men no significant links emerged between gender-related sexual attitudes and affect through sexual autonomy, sexual body esteem, or approach / avoidance motives for sex. The results thus show that the endorsement of more traditional gender-related sexual attitudes is associated with self-related sexual cognitions and emotions in a way that indicates negative implications specifically for women.

The first hypothesis, that women’s stronger endorsement of traditional gender-related sexual attitudes would be related to lower levels of positive and higher levels of negative sex-related affect through lowered sexual autonomy, sexual body esteem, and approach motives for sex, as well as heightened avoidance motives for sex, was partially confirmed. Traditional gender-related sexual attitudes appeared to be related to decreased positive and increased negative affect namely through decreased sexual autonomy and increased avoidance motives for sex. For women, increased scores on gender-related sexual attitudes were related to decreased sexual autonomy, which was in turn related to increased negative affect. Considering that gender-related sexual norms value sexual modesty and submission in women (Sanchez et al. 2012a), this finding is very tenable. It also matches findings from an earlier study on sexual autonomy (Kiefer and Sanchez 2007). The discrepant findings between men and women in sexual autonomy indicate that this may be a key factor, which sustains sexual double standards in sexual and romantic interactions.

Additionally, we found that stronger endorsement of gender-related sexual attitudes among women was related to decreased positive and increased negative sex-related affect through avoidance motives for sex. These findings corroborate the notion that traditional gender-related sexual attitudes are successful in producing normative rather than individually desired behavior. If sex is pursued for other than personal reasons, this may harm young adults in more than one way; it may be detrimental to protective sexual behaviors, but may also encourage sexual coercion and violence. Occasionally having sex prompted by avoidance motives would probably not be too harmful. However, when it becomes a pattern this may be bad for well-being on both a personal and relationship level (Impett et al. 2005).

Among women, decreased sexual body esteem was related to the stronger endorsement of gender-related sexual attitudes. The relationship between sexual body esteem and positive and negative affect did not however reach significance. The relationship between gender-related sexual attitudes and sexual body esteem might be explained by sexual self-objectification. There is evidence that sexual objectification, either experienced as a result of certain (cultural or media) cues or as an internalized perception, increases self-consciousness about body image during physical intimacy, which puts adolescents (and notably girls) at risk of being preoccupied with how they appear during sex rather than appreciating how their bodies feel (Aubrey 2007; Fredrickson and Roberts 1997; Vandenbosch and Eggermont 2014). Translated to the context of our study, this implies that relatively gender-traditional women may feel more self-conscious about their bodies. However, our expectation that this would, in turn, be related to less positive and more negative sex-related affect was not confirmed. Although sexual body esteem might still be a relevant factor to investigate when examining sexual double standards, it does not seem to provide any additional insight into the link between gender-related sexual attitudes and sex-related affect in our study.

As to why sexual body esteem was not significantly related to positive or negative affect among women, we would suggest that body esteem is possibly, in comparison to the other cognitive mediators, less directly related to actual sexual behavior (sexual autonomy as well as approach and avoidance items all included references to actual sexual behaviors). On a similar note, sexual body esteem could arguably comprise both cognitive and affective components, possibly obscuring the relationships between variables. In this regard, future researchers are advised to select mediators and scales even more carefully, being aware that there may be several different “types” of cognitive mediators and that measured concepts may not be clearly either cognitive or affective, but rather incorporate both.

Our second hypothesis—that men’s stronger endorsement of traditional gender-related sexual attitudes would be related to higher levels of positive and lower levels of negative sex-related affect through heightened sexual autonomy and approach motives for sex, but lower avoidance motives for sex—is rejected. In this respect, it is interesting to note that although we see many gendered differences in the relationships between variables, we see almost no differences between men and women when comparing their mean scores. Our findings confirm that, although traditional gender-related sexual attitudes may be endorsed to roughly the same extent among women and men, their impact on self-related sexual cognitions and emotions (as measured in our study) is only seen among women. These women, characterized by a relatively stronger belief in traditional gender norms, could be regarded as being at particular risk of negative sexual and mental health outcomes through a detrimental psychosexual pattern of self-related sexual cognitions and emotions.

However, this does not necessarily mean that there are no (positive or negative) effects among men. It is possible that the concepts measured (i.e., the cognitive and emotional factors) in our study were in retrospect less well suited to a sample of men. It may be that stronger traditional gender-related sexual attitudes are unrelated to affect, but are more readily visible in other areas. For example, research has shown that effects of traditional masculinity may hamper social and emotional development (Brooks 2001) as well as self-disclosure and responsiveness (Marshall 2010). It could also be that stronger traditional gender-related sexual attitudes are related to affect for men, but that these are mediated through other cognitive factors, such as performance failure (Clarke et al. 2015). We would therefore argue that future studies should investigate other cognitive aspects (such as reduced openness, responsiveness, connectedness, and performance pressure) in relation to gender-related sexual attitudes, which might be more relevant among men.

Lastly, although approach motives (e.g., the message that sex is a means of satisfying one’s own needs or of improving intimacy in a relationship) were related to positive and negative sex-related affect in the expected ways, the relationship between gender-related sexual attitudes and affect was not mediated by approach motives for either men or women. One explanation for the absence of mediation by approach motives is that they are heavily emphasized in the comprehensive sexual education that Dutch schools offer; a central message in that education is that you should “only do what you want to do,” and the curriculum is highly “sex-positive” (Ferguson et al. 2008). It could be that this message is so instilled in Dutch young people that approach motives are a given, rendering them fairly independent of the individual’s gender-related sexual attitudes, at least in this particular sample of predominantly white native Dutch young people. Additionally, the absence of gender differences as regards approach motivations and their link with the other variables in our model suggests that approach motives for sex might be regularly emphasized in modern sexual socialization for both men and women. Avoidance motives for sex (e.g., the message that one should be sexually compliant and that sex is a means of avoiding conflict), on the other hand, might be emphasized only in the sexual socialization of women. Because approach and avoidance tendencies are at least partially learnt through gendered socialization (Tolman 2006), it would be interesting for future research to look more closely at their effects on gender-related sexual attitudes and sexual motives.

### Limitations and Future Research Directions

There are some limitations to our study. First, the nature of the measurement instrument SASSY may have yielded socially desirable responses. However, because the questionnaire was completed online, presumably in the comfort of the respondent’s own home, we believe that this risk was reduced (Spijkerman et al. 2009). Nevertheless, the possibility of socially desirable responding can never be ruled out entirely when using explicit self-report questionnaire surveys. Another hint in this direction may be the relatively low scores on endorsement of traditional gender-related sexual attitudes in our sample. These results echo those of a previous study among adolescents in which the same instrument was used (Emmerink et al. 2016), and they also match findings from other explicit (questionnaire-based) surveys of gender-related sexual attitudes (Bordini and Sperb 2013). However, because the scale scores in our study showed sufficient variance, the pattern of scores indicates both a rather high level of egalitarianism in the majority of the sample and a smaller group of people for whom traditional sexual attitudes are still dominant. This also matches findings from a relatively recent review study (using research published in *Sex Roles*), which concludes that “heterosexual dating among young adults in the U. S. remains highly gender-typed in terms of cultural scripts and interpersonal scripts” (Eaton and Rose 2011, p. 843). In future studies we will aim to investigate different types of measurements further, seeking to compare scores on self-report explicit measurements to scores on newly designed implicit measurements (specifically an Implicit Association Test to assess gender-related sexual attitudes; see Sakaluk and Milhausen 2012 for pioneering work on this topic). Making use of this new implicit type of measurement could possibly reduce (socially desirable) response tendencies in future studies. Moreover, it might provide additional insight into the continued existence of the sexual double standard because it may exist both on a conscious (e.g., as an attitude) and non-conscious (e.g., as a stereotype) level.

Second, our study was cross-sectional in nature. It should therefore be noted that no causal inferences can be made based on our study. In the model that we tested, gender-related sexual attitudes were used as the indicator, the cognitive factors as mediators, and affect as the outcome. However, the correlates examined in our study are assuredly in continuous interaction with each other through people’s lived experiences. For example, cognitions are able to influence emotions, but emotions are also able to influence cognitions. Similarly, activated cognitions and emotions in sexual situations may be both the result and the instigator of sexual behavior. Gender-related sexual attitudes can therefore be presumed to change over time as a result of the interactions among cognitions, emotions, and choices in and outcomes of sexual behavior. This process can certainly be seen as an example of “doing gender” (West and Zimmerman 1987, p. 126). It would be very interesting for future research to investigate these influential processes further using Deaux and Major’s (1987) interactive model of gender-related behavior, which encompasses both stability and flexibility in gender-related behavior and stresses display rather than acquisition of gendered behavior.

Third, a further limitation of our study was our use of a convenience sample, which consisted of predominantly white ethnically Dutch individuals. This hampers generalizability to the culture of the Netherlands as a whole. However, even within this predominantly white sample, despite Dutch culture being relatively open-minded about sexuality and the Dutch government supporting comprehensive sex education (Dodge et al. 2005), we still find evidence of traditional gender-related sexual attitudes and their negative effects. We may therefore conclude that merely being open-minded about sexuality and delivering comprehensive sexual education do not mean that normative gender roles in line with the sexual double standard do not exist. These findings support the notion that the sexual double standard still exerts a continued influence and that the problem might even exist in a more covert form. Because the shaping of gender-related sexual attitudes is so culture- and context-dependent (Bordini and Sperb 2013; Deaux and Major 1987; West and Zimmerman 1987), future studies might fruitfully investigate how different cultural beliefs concerning sexuality within the multicultural Dutch society are related to each other and how they relate to sexual well-being in light of the sexual double standard. As a consequence of the predominantly white Dutch sample, the results of our study may even paint a rosier picture compared to a study which used a sample that more adequately reflected multicultural Dutch society. An earlier study which assessed the psychosexual correlates of sexual double standard endorsement found relatively strong gender traditional attitudes among non-native Dutch individuals and religious individuals (Emmerink et al. 2016), who were not well represented in the present study.

A final limitation, which has been discussed at length previously, is the possibility that we may have found results among the women in the sample but not among the men because of the nature of the concepts measured in our study. Future research should try to include more cognitive factors related to notions of traditional masculinity.

Although not assessed in the present study, sexual desire is an additional factor of interest (for both theoretical and practical purposes) that could be discussed in respect to our findings on sexual autonomy as well as approach and avoidance (Impett and Tolman 2006; Tolman 1991; Tolman and Tolman 2009). We argue that women, who are very traditional in their gender-related sexual attitudes and who are presumably less sexually autonomous and have less approach and more avoidance motives for sex, might suffer from low sexual desire (Sanchez et al. 2006). We presume that this may be the case because they are not anticipating a sexual encounter as much (Vannier and O’Sullivan 2010). We know that instances of sexual compliance (where individuals agree to sex from an avoidance motivation) are regarded as relatively unexpected. Moreover, it may take longer for these individuals to become aroused and interested in sexual activity (Vannier and O’Sullivan 2010). Because women who endorse traditional gender patterns may have internalized the idea that sexual desire is relatively inappropriate for them (Impett and Tolman 2006), they might also be less likely to anticipate sexual situations and in turn experience lower sexual desire as well as less positive, and more negative, affect. This might also partially explain why women tend to experience more sexual arousal difficulties than men (Laumann et al. 1999). This idea is corroborated by a study which found approach and avoidance goals to be related to increased and decreased desire, respectively, which in turn led to more and less sexual satisfaction, respectively (Muise et al. 2013).

### Practice Implications

We would draw attention to the implications of the gendered effects that were found for sexual autonomy. Because adequate sexual autonomy is needed in order to achieve satisfactory sexual functioning in both women and men (Kiefer et al. 2006), we argue that sexual autonomy deserves explicit attention in sex-positive and gender-transformative sexuality education—something which has been shown to be a vital resource for achieving more gender-equitable and healthy relationships (Grose et al. 2014; Haberland 2015; Lamb and Peterson 2012). Tailored educational programs may bring about further change in this respect. In the United States, it has been shown that sex-positive and gender-transformative education is still rare (Fine and McClelland 2006). Sex education in the United Kingdom has been criticized for similar reasons (Ingham 2005). Although there are notable differences between sex education in the United States and the Netherlands (Ferguson et al. 2008), the present study appears to imply that there are still points for improvement, including in the Netherlands. One task of Dutch sexual education would seem to be to narrow the gender gap in sexual autonomy and address issues of sexual body esteem and avoidance sexual motives especially among girls and women. The results of our study specifically warrant early identification of girls and women who struggle with the pressure to conform to gender expectations and who might later be at risk for the negative implications of that pressure.

### Conclusions

Our study illustrates the relationship between gender-related sexual attitudes, cognitions, and emotions, variables that are presumed to be in continuous interaction with each other as a result of “doing gender” (West and Zimmerman 1987). Our research design has enabled us to shed light on cognitive and emotional processes with regard to gender-related sexual attitudes, without necessarily implying one-directional causal relationships. We conclude that, as a result of negative cognitions associated with the endorsement of traditional gender norms, women in particular are at risk of experiencing negative emotional outcomes in the sexual context. We argue that sexual cognitions and emotions deserve explicit attention in sex-positive and gender-transformative sexuality education, which has proven to be a vital resource for achieving increased gender equity in sexual and romantic relationships.

## Electronic supplementary material

Below is the link to the electronic supplementary material.

ESM 1 (DOCX 32 kb)
