# Supplementary material for: The Relationship Between Endorsement of the Sexual Double Standard and Sexual Cognitions and Emotions
Source: Sex Roles. 2016 Apr 8;75(7):363–76. doi: 10.1007/s11199-016-0616-z (PMC5023751; doi:10.1007/s11199-016-0616-z)
Supplement: Supplementary file 1 — (DOCX 32 kb) [file 11199_2016_616_MOESM1_ESM.docx]

Online supplement for Emmerink, P. M. J., van den Eijnden, R. J. J. M., Vanwesenbeeck, I., & ter Bogt, T. F. M. (2016). The relationship between endorsement of the sexual double standard and sexual cognitions and emotions. *Sex Roles*. Peggy M.J. Emmerink, Utrecht University, E-mail: [p.m.j.emmerink@uu.nl](mailto:p.m.j.emmerink@uu.nl).

**Scale for the Assessment of Sexual Standards among Youth (SASSY)**

| Item # | English Language Item Wording |
| --- | --- |
| 1 | Once a boy is sexually aroused, a girl cannot really refuse sex anymore. |
| 2 | I think that a girl who takes the initiative in sex is pushy. |
| 3 | I think it is more appropriate for a boy than for a girl to date different people at the same time. |
| 4 | Girls should act in a more reserved way concerning sex than boys. |
| 5 | I think it is more appropriate for a boy than for a girl to have sex without love. |
| 6 | A boy should be more knowledgeable about sex than a girl. |
| 7 | I think sex is less important for girls than for boys. |
| 8 | I think it is normal for boys to take the dominant role in sex. |
| 9 | I think sexually explicit talk is more acceptable for a boy than for a girl. |
| 10 | Sometimes a boy should apply some pressure to a girl to get what he wants sexually. |
| 11 | It is more important for a girl to keep her virginity until marriage than it is for a boy. |
| 12 | Boys are more entitled to sexual pleasure than girls. |
| 13 | It is not becoming for a girl to have unusual sexual desires. |
| 14 | Sex is more important for boys than for girls. |
| 15 | It is more important for a girl to look attractive than it is for a boy. |
| 16 | Boys and girls want completely different things in sex. |
| 17 | I think cheating is to be expected more from boys than from girls. |
| 18 | I think it is important for a boy to act as if he is sexually active, even if it is not true. |
| 19 | I think it is more appropriate for a boy than for a girl to masturbate frequently. |

*Note.* The instrument was administered in the Dutch language.

| Item # | Dutch Language Item Wording |
| --- | --- |
| 1 | Als een jongen eenmaal seksueel opgewonden is, mag een meisje eigenlijk geen seks meer weigeren. |
| 2 | Ik vind het opdringerig staan als een meisje het initiatief neemt tot seks. |
| 3 | Ik vind het meer bij een jongen passen dan bij een meisje om verschillende scharrels tegelijkertijd te hebben. |
| 4 | Meisjes moeten zich op het gebied van seks terughoudender gedragen dan jongens. |
| 5 | Ik vind het meer bij een jongen passen dan bij een meisje om seks te hebben zonder gevoel. |
| 6 | Een jongen hoort meer van seks af te weten dan een meisje. |
| 7 | Ik vind seks voor een meisje minder belangrijk dan voor een jongen. |
| 8 | Ik vind het normaal dat een jongen de dominante rol neemt in seks. |
| 9 | Schunnige dingen zeggen over seks schokt mij eerder van een meisje dan van een jongen. |
| 10 | Soms moet een jongen een beetje druk uitoefenen op een meisje om op seksueel gebied te krijgen wat hij wil. |
| 11 | Voor een meisje is het belangrijker om maagd te blijven tot ze trouwt dan voor een jongen. |
| 12 | Een jongen heeft meer recht op seksueel genot dan een meisje. |
| 13 | Een meisje hoort geen ongewone verlangens te hebben op seksueel gebied. |
| 14 | Voor jongens is seks belangrijker dan voor meisjes. |
| 15 | Voor een meisje is het belangrijker om er aantrekkelijk uit te zien dan voor een jongen. |
| 16 | Jongens en meisjes willen heel verschillende dingen in seks. |
| 17 | Ik vind het van jongens meer te verwachten dat ze vreemdgaan dan van meisjes. |
| 18 | Ik vind het belangrijk dat een jongen zich seksueel actief voordoet ook al is hij dat niet. |
| 19 | Ik vind het meer bij een jongen passen dan bij een meisje om vaak te masturberen. |

*Note.* The instrument was administered in the Dutch language.
